# The Level and Development of Unemployment before Disability Retirement: A Retrospective Study of Finnish Disability Retirees and Their Controls

**DOI:** 10.3390/ijerph17051756

**Published:** 2020-03-08

**Authors:** Mikko Laaksonen, Jenni Blomgren

**Affiliations:** 1 Finnish Centre for Pensions (ETK-Eläketurvakeskus), 00065 Helsinki, Finland; 2 The Social Insurance Institution of Finland (KELA), 00520 Helsinki, Finland

**Keywords:** unemployment, disability retirement, work ability, socioeconomic

## Abstract

A weakening work ability may lead to a higher risk of gradual exclusion from working life, which may be manifested in increasing levels of unemployment. This study examined development of unemployment prior to disability retirement by educational level and occupational class in different diagnostic groups. The study population comprised 70% of Finnish residents aged 25–64 years who retired due to disability in 2011–2015 (n = 54,387). Growth curve models were used to analyze the level and development of pre-retirement unemployment among the retirees due to mental disorders, musculoskeletal diseases and all other somatic diseases and their gender- and age-matched controls drawn from the non-retired population. During six pre-retirement years, disability retirees had on average 39 annual excess unemployment days compared to their non-retiring controls. Excess unemployment was particularly high among those retiring due to mental disorders. On average, unemployment increased by 5.5 days per each year of approaching disability retirement, after controlling for aging and secular trends. The increase was largest among those who retired due to mental disorders. Excess unemployment was higher among the less educated and among manual workers, in particular among those retiring due to mental disorders or somatic diseases other than musculoskeletal diseases. Increased efforts to maintain and improve work ability among the unemployed is crucial in diminishing disability retirement at the population level. As the level of unemployment is elevated already several years before disability retirement, work ability problems among the unemployed should be tackled in the early stages.

## 1. Introduction

High numbers of working-age persons on disability benefits is acknowledged to be a severe problem for the sustainability of modern welfare societies. In the OECD countries, about 6 percent of the working-age population receive disability benefits [1]. In order to understand the mechanisms of disability development and to recognize possibilities for timely prevention, it is important to gain more knowledge about the pathways to permanent disability. More evidence is needed on the process and predictors of weakening work ability among those on the path to disability retirement compared to those who are able to remain active in working life.

Disability pension is often granted after a long period of gradually weakening work ability. Previous studies have shown that sickness absence levels are increased already several years before disability retirement [2,3]. Furthermore, weakening work ability may lead to a higher risk of gradual exclusion from working life, which may be manifested in increasing levels of unemployment. In accordance with this, previous studies have shown that unemployment is associated with increased risk of disability retirement [4,5]. In a Norwegian study losing one’s job more than doubled the risk of permanent disability retirement [6]. Unemployment could have an instantaneous triggering effect on applying for a disability pension or its effect could develop over the longer term, if one’s work ability gets worse during unemployment [7,8]. However, previous studies have not examined changes in unemployment before disability retirement.

Disability retirement is strongly associated with one’s socioeconomic status. Among the poorly educated and manual workers, the risk of disability retirement due to musculoskeletal diseases is particularly high, whereas retirement due to mental disorders shows smaller socioeconomic differences [9,10]. Moreover, unemployment is more common among people with a lower education or occupational class [11]. It is therefore plausible that the role of unemployment in the disability retirement process may vary according to one’s socioeconomic status.

The aim of this study was to assess the role of unemployment in the disability retirement process in a retrospective setting using nationally representative register-based data. We calculated the development of the yearly number of unemployment days during six years before disability retirement among the retirees and their gender- and age-matched controls. As unemployment is more strongly related to mental than physical health problems [12,13], we separately examined those who retired due to mental disorders, musculoskeletal diseases and other somatic diseases. Furthermore, we examined whether the level and development of pre-retirement unemployment differs by socioeconomic status.

## 2. Materials and Methods

### 2.1. Participants and Procedure

In the Finnish social security system, all working-age residents are insured against the loss of work ability due to impaired health. For short-term work disability lasting up to one year, compensation is paid in the form of a sickness allowance. If one’s work disability continues for more than one year, a disability pension may be granted [14]. The disability pension can be paid from the earnings-related pension scheme for those who have accrued pension by working or from the national pension scheme which secures one’s subsistence when one has no earnings-related pension or when it is very small. The earnings-related pension schemes applies to persons aged 18–62 years whereas the national disability pension scheme covers those aged 16–64 years. After these age limits, the pensions are transferred to old-age pensions and new disability pensions will not be granted [15]. 

Due to data protection regulations, the current study was based on a 70% sample, drawn at random, from the entire Finnish population, utilizing the population data file of the Social Insurance Institution of Finland. From this sample, we identified all persons aged 25–64 who received a new full-time disability pension from either one of the pension schemes in 2011–2015. The information was based on the pension registers of the Finnish Centre for Pensions and the Social Insurance Institution of Finland. The number of disability retirees in the dataset was 54,387.

The retirees were divided into three groups based on primary diagnosis of their pension. The groups were mental and behavioral disorders (ICD-10 codes F00–F99, n = 16,658), musculoskeletal diseases (M00–M99, n = 16,582), and all other somatic diseases (n = 21,147). In the group of other somatic diseases, diseases of the nervous system, cardiovascular diseases, neoplasms and injuries, each account for one fourth of the cases. Within these diagnostic groups, three controls matched for gender and age (birth year) were randomly drawn for each of the disability retirees from the non-retired population in the above-mentioned dataset (n = 163,161).

Unemployment was measured through register-based records of unemployment benefits that include all unemployment spells that exceed the waiting time of five working days. For each disability retiree, the number of unemployment days was calculated in one-year (365 days) intervals, counting backwards from the retirement, up to six years before the start of the pension. Thus, the earliest measurement of unemployment for those who retired in 2011 was from the year 2005 and for those who retired in 2015 from the year 2009. For the controls, the unemployment days were recorded backwards from the 1st of July of the corresponding year. 

We used two complementary indicators of socioeconomic position derived from Statistics Finland: educational level and occupational social class. Educational level was classified into primary education or no qualifications, secondary, lower tertiary and higher tertiary education. Occupational class was classified into manual workers, lower non-manual employees (such as nurses or office clerks), upper non-manual employees (such as dentists or lawyers), entrepreneurs (including self-employed and owners of companies with salaried employees), and others, consisting mainly of unemployed persons, students and those who lacked information on their occupation. The measurement was taken from the year preceding disability retirement. For those classified as ‘others’, the most recent occupational class from the preceding five years was used, if found. 

### 2.2. Statistical Methods

We first described the number of unemployment days in one-year intervals during the six years preceding disability retirement among retirees in the three diagnostic categories and among the controls. We then used growth curve models to analyze the level of unemployment between the retirees and the controls, and to calculate the average yearly change of unemployment days during the years preceding disability retirement among the eventual retirees. For simplicity, linear growth was assumed in random-coefficient models with a random slope of time to retirement [16]. The analyses were conducted separately in the categories of gender, age and the socioeconomic variables and for each of the three diagnostic categories. Statistical significance was judged from the 95% confidence intervals. As the general level of unemployment may vary according to macroeconomic trends, and the amount of unemployment days may increase due to the aging of the study population, we adjusted for calendar-year and time-varying age. In analyses by educational level and socioeconomic status, gender was adjusted to control for socioeconomic differences between men and women. The deviating final year preceding disability retirement (see below) was excluded from the models. The analyses were conducted using Stata 14.2.

### 2.3. Ethics Statement

We adhered to the ethical standards of The Finnish Advisory Board on Research Integrity when we collected, analyzed and reported the data. In Finland, ethics approval is not required for studies based solely on register data [17].

## 3. Results

### 3.1. Descriptive Characteristics of the Disability Retirees and the Controls

The distributions of the disability retirees and the controls by gender, age and the socioeconomic characteristics are shown in Table 1. Slightly more than half of the disability retirees were men, and the retirees were concentrated in the older age groups. Half of the disability retirees had a secondary education and two thirds had been working in manual or lower non-manual occupations. However, there were large differences in these distributions when examined by the diagnosis underlying the disability pension. Those who retired due to mental disorders were more often women while those retiring due to other diagnoses were predominantly men. Retirees due to mental disorders were clearly younger than other retirees. The distribution of educational level was broadly similar in all diagnostic groups, but those retiring due to musculoskeletal diseases or other diseases were more often manual workers than those retiring due to mental disorders. Especially among those who retired due to mental disorders, there was a large group whose occupational social class could not be determined. 

Due to the matching, the controls corresponded to the retirees by gender and age, but they were more often tertiary educated and upper non-manual employees than disability retirees (Table 1). 

Table 1 also presents the mean number of unemployment days (and standard errors) by the study variables among disability retirees and the controls (i.e., the general population). Data from the second last year before disability retirement is given. On average, the disability retirees had 94, and the controls, 39 unemployment days. Men had slightly more unemployment days than women. Among disability retirees, the number of unemployment days slightly decreased by age, whereas the controls aged 55–64 years had more unemployment days than the younger. The number of unemployment days decreased strongly with increasing education and occupational social class. Among those with ‘other’ or ‘unknown’ occupational class, the number of unemployment days was extremely high. As occupational class was measured retrospectively from the preceding five years, this group consists of people who have been outside the labor market for a fairly long time. 

### 3.2. Trends of Unemployment Before Disability Retirement

**Figure 1:**
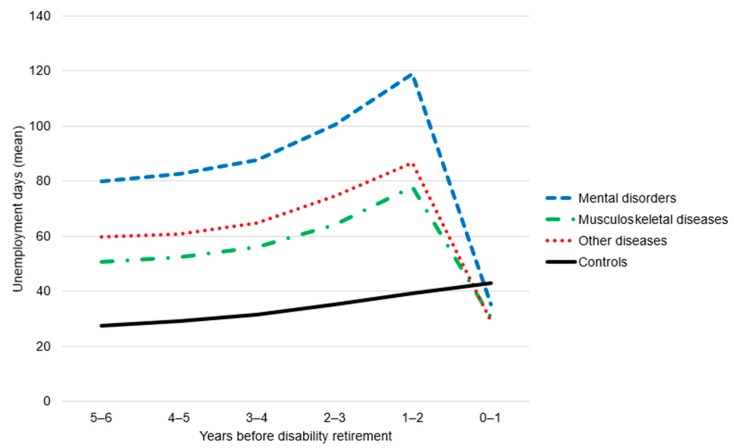
Annual number of unemployment days (mean) during the six years preceding disability retirement among disability retirees in the three diagnostic groups and their age- and gender-matched control.

Figure 1 shows the average number of annual unemployment days during the six years preceding disability retirement in the three diagnostic groups of disability retirees and among the controls. During the pre-retirement years, the average number of unemployment days was clearly higher among the disability retirees than among the controls. Furthermore, those who retired due to mental disorders had far more unemployment days than those who retired due to musculoskeletal diseases or other somatic diseases. In all three diagnostic groups, the number of unemployment days increased when the date of disability retirement approached. However, during the final year preceding disability retirement, the number of unemployment days collapsed because the applicants normally spend the last year before their disability pension on a sickness allowance, which is the primary benefit for a weakening work ability.

The average number of unemployment days increased also in the control group during the observation period. Despite the differences in gender and age distributions, unemployment rates among the controls were fairly equal in all diagnostic groups, and, for the sake of clarity, they were pooled for the figure. Yet, among the controls for those who retired due to musculoskeletal diseases or other somatic diseases, unemployment increased slightly more than among the controls for the retirees due to mental disorders (see Table 2).

### 3.3. Increase in Unemployment Controlling for the Effects of Calendar Time and Aging

The trend of increasing unemployment with approaching retirement may be confounded by changes in the general unemployment rate over time. In Finland, unemployment increased from 2008 to 2015, that is, during most of our study period [11]. In addition, the study population also became six years older during the follow-up. The increasing number of unemployment days in the control group suggests that an increase in unemployment rate over time and/or aging of the study population may lie behind these changes. Table 2 shows that, per one year of approaching disability retirement, unemployment increased by 7.6 days among the disability retirees and by 3.0 days among the controls. The increase was highest among those who retired due to mental disorders. When calendar year and age were adjusted, the trend of increasing unemployment in the control group was eliminated. Among the disability retirees, the increase in unemployment reduced to 5.5 days per year. The adjustments had a somewhat stronger effect among those who retired due to musculoskeletal diseases or other somatic diseases than among those who retired due to mental disorders. In the following analyses, calendar year and age have been controlled.

### 3.4. Excess Unemployment among Disability Retirees Compared to the Controls

During the pre-retirement years, disability retirees had, on average, 38.6 more annual unemployment days than the controls (Table 3). Those who retired due to mental disorders had nearly 60 excess unemployment days while the difference was smaller among those who retired due to musculoskeletal diseases or other somatic diseases. Thus, compared to the control group (the general population) with an average of 33 annual unemployment days, pre-retirement unemployment was roughly twofold among all disability retirees and almost threefold among those who retired due to mental disorders.

Excess unemployment among the retirees was larger among men than among women, except among those who retired due to musculoskeletal diseases. Among those who retired due to mental disorders, those in the youngest and the oldest age group had relatively little excess unemployment days compared to those in the middle age categories. In the other diagnostics groups, the excess unemployment was smallest in the oldest age group.

Those with a primary or secondary education had more excess unemployment than those with a tertiary education. However, this association was not found among those who retired due to musculoskeletal diseases. The overall differences between manual workers and both groups of non-manual employees were small but showed diverging associations in different diagnostic groups: especially among those retiring due to musculoskeletal diseases, excess unemployment was clearly smaller among manual workers. However, entrepreneurs had the least and those with ‘other’ or ‘unknown’ occupational class, the most excess unemployment in all diagnostic groups.

### 3.5. Increase in Unemployment among Disability Retirees

Among those who retired due to mental disorders, unemployment increased by 8.0 days per each year of approaching disability retirement. Among the retirees due to musculoskeletal diseases, the increase was, on average, 3.8 days, and among those retiring due to other diseases, 3.3 days (Table 4). In the subgroups of gender and age, as well as in different socioeconomic groups, the increase in pre-retirement unemployment was generally rather similar and the differences between the categories of each variable did not reach statistical significance for the most part. Among those who retired due to musculoskeletal diseases or other somatic diseases, there was no statistically significant increase in unemployment in the two youngest age groups and among those with a tertiary education. Among manual workers, the increase in pre-retirement unemployment was somewhat larger than in other groups, in particular, as compared to the entrepreneurs.

## 4. Discussion

We examined the level and development of unemployment during the years preceding disability retirement using register-based data on Finnish disability retirees in 2011–2015. The study showed that during the six pre-retirement years, future disability retirees had clearly more days of unemployment than the control group of the same gender and age. Unemployment was especially common among those retiring due to mental disorders. On average, the number of annual unemployment days increased when disability retirement approached, and this increase was largest among those retiring due to mental disorders. The pre-retirement increase was partly explained by secular trends in unemployment and the aging of the study population, but most of the increase was genuinely related to the approaching disability retirement.

A precondition for disability retirement is a long-term decrease in one’s work ability. The high overall level and increase in unemployment as disability retirement approaches is likely to be a marker of poor and constantly deteriorating health. We are not aware of any previous studies examining long-term changes in unemployment before disability retirement, but there is evidence that while poor health may increase the risk of unemployment, unemployment may also trigger health problems [7,8,18,19]. Furthermore, unemployment and poor health are likely to have a self-reinforcing relationship so that those with some health problems have an increased risk of unemployment, and unemployment, in turn, further deteriorates one’s health and work ability, which may again weaken one’s employment opportunities [20,21]. As the criteria for a disability pension are rather strict, requiring a continuous reduction of work ability for at least one year, health problems may increase unemployment before retirement on a disability pension. Furthermore, the experience of unemployment may also cause persons with health problems to apply for a disability pension. In a recent Norwegian study, a large proportion of disability insurance claims could be directly attributed to job loss or other adverse shocks to employment opportunities [6].

Unemployment histories were more extensive and the increase in unemployment steeper among those who retired due to mental health problems. This is consistent with the findings that unemployment is more strongly associated with mental than physical health [12,13]. Mental health problems typically emerge at a relatively young age, which may lead to more fragile employment histories. Furthermore, the prognosis of mental disorders is often uncertain [22,23]. It is therefore understandable that the progression of an illness is monitored for some time before a disability pension is granted. This waiting time may consist of periods of unemployment, working and receiving other social benefits. Only a small minority of depressive disorders eventually lead to disability retirement [24,25].

Considering all disability retirees, excess unemployment was more pronounced among men and in the younger age groups. The excess was also slightly higher among those with a primary or secondary education than among those with a tertiary education. The differences between manual workers and upper and lower non-manual employees were small. However, the excess unemployment is also affected by the level of unemployment in the corresponding control group. Thus, even if the number of excess unemployment days among manual workers and non-manual employees was fairly similar, manual worker retirees, nevertheless, have more unemployment days as their overall level of unemployment is higher.

Furthermore, socioeconomic differences in excess unemployment varied across diagnostic groups. Larger excess unemployment in the lower socioeconomic groups was seen among those who retired due to mental disorders and somatic diseases other than musculoskeletal diseases. The differences were small among those who retired due to musculoskeletal diseases. Non-manual employees retiring due to musculoskeletal diseases had larger excess unemployment than manual workers. Most of those who retired due to musculoskeletal diseases were manual workers or lower non-manual employees; few had a tertiary education. Physical workload and poor ergonomics are important risk factors for musculoskeletal disorders [26,27] and disability retirement due to musculoskeletal diseases [28,29]. The absence of these risk factors is likely to diminish the role of unemployment as a route to disability retirement due to musculoskeletal diseases, especially among manual workers.

The differences between sociodemographic groups in increasing unemployment when disability retirement approached were small, and the differences between these groups rarely reached statistical significance. Thus, there was more variation in the initial level and propensity of unemployment between sociodemographic groups than in changes in the burden of unemployment in these groups with approaching retirement. In different socioeconomic groups, the causes behind disability retirement due to mental disorders may be different. Work-related stress may contribute to disability retirement in occupations with high mental demands [30]. This may explain why excess unemployment due to mental disorders was less prevalent among retirees from the higher occupational classes.

### Methodological Considerations

We used a large sample (70% of the Finnish population) drawn at random from national registers. Thus, the sample was fully representative of Finnish disability retirees and their controls. Register-based information of disability retirement was linked to register data on unemployment benefits and on sociodemographic covariates. Register-based data are considered highly reliable, with no self-report bias or loss to follow-up and with very little missing information. The Finnish disability pension system is universal and applies to all people, irrespective of their labor market status. The same rule applies to the employed and unemployed applicants. The results may not be generalizable to countries where the requirements for being allowed unemployment or disability benefits and the financial incentives for applying for these benefits differ.

Due to the detailed nature of the registers, we were able to measure both disability retirement and unemployment using date-specific information. The analyses included disability retirees from five calendar years, which allowed us to separate the effect of secular trends and aging from the true increase of unemployment when disability retirement approached. However, the measure of unemployment was based on benefit receipt, and other social security benefits may hinder the receipt of unemployment benefits. This is clear, particularly during the last year before disability retirement, when many disability pension applicants receive sickness allowance.

Unemployment was measured as the number of unemployment days in one-year intervals. The measure is rather crude, and there is much variation within the disability retirees in their past unemployment history. We conducted sensitivity analyses using different cut-off points for a dichotomous measure of unemployment, but the general conclusions were not affected. For example, using any employment or unemployment of at least 90 days, the results were very similar to those obtained using a continuous measurement of unemployment.

## 5. Conclusions

The number of unemployment days increased slightly as disability retirement approached, but this increase was modest compared to the high overall level of unemployment among the disability retirees. Unemployment was most common among those who retired due to mental disorders, especially if they came from lower socioeconomic groups. Increased efforts to maintain and improve work ability among the unemployed, for example, by means of rehabilitation [31,32] is crucial in diminishing disability retirement at the population level. As the level of unemployment is elevated already several years before disability retirement, work ability problems among the unemployed should be tackled in the early stages.

## Figures and Tables

**Table 1:** Distribution of the study variables (%) among disability retirees (separated by the diagnosis of disability pension) and their gender- and age-matched controls (i.e., the general population) and the mean number of unemployment days (standard error) among the disability retirees and the controls during the second last year before disability retirement.

	Distribution of the Study Variables (%)					Unemployment Days, Mean (s.e.) ^a^	
	All Disability Retirees	By Diagnosis of Disability Pension			Controls ^b^	Disability Retirees	Controls
		Mental Disorders	Musculoskeletal Diseases	Other Diagnoses			
Gender							
Men	53	47	51	60	53	100.0 (0.85)	42.2 (0.35)
Women	47	53	49	40	47	86.7 (0.87)	36.1 (0.35)
Age							
25–34 years	10	22	3	6	10	100.2 (1.91)	32.6 (0.68)
35–44 years	13	21	8	11	13	102.6 (1.71)	33.0 (0.62)
45–54 years	28	30	25	30	28	95.1 (1.15)	32.8 (0.42)
55–64 years	49	28	64	54	49	89.5 (0.87)	46.3 (0.39)
Educational level							
Primary	29	26	31	29	18	110.6 (1.21)	59.8 (0.70)
Secondary	53	49	58	51	43	95.4 (0.84)	45.3 (0.40)
Lower tertiary	15	18	10	16	27	65.0 (1.39)	25.2 (0.39)
Higher tertiary	4	6	1	4	12	60.1 (2.65)	18.6 (0.50)
Occupational class							
Manual workers	39	27	49	40	28	84.0 (0.91)	53.4 (0.51)
Lower non-manual employees	27	28	27	26	32	63.1 (1.01)	26.7 (0.36)
Upper non-manual employees	8	11	4	9	20	58.9 (1.79)	16.6 (0.36)
Entrepreneurs	11	9	12	12	12	38.3 (1.32)	9.6 (0.37)
Other or unknown	16	25	9	14	7	223.8 (1.70)	151.1 (1.49)
All	100	100	100	100	100	93.8 (0.61)	39.4 (0.25)
N	54,387	16,658	16,582	21,147	163,161		

^a^ The mean number of unemployment days during the second last year before disability retirement and among the controls during a corresponding time period; ^b^ The controls were drawn separately in the three diagnostic groups but have been collapsed together for this table.

**Table 2:** The effect of adjusting for calendar year and age on the increasing number of unemployment days before disability retirement. Mean increase in the number of unemployment days per one year of approaching disability retirement (95% confidence intervals). The last year before disability retirement omitted.

	Unadjusted	Adjusted for Calendar Year and Age
Disability retirees		
Mental disorders	9.5 (9.0–10.1)	8.0 (6.6–9.3)
Musculoskeletal diseases	6.6 (6.1–7.0)	3.8 (2.6–5.0)
Other diseases	6.8 (6.3–7.2)	3.3 (2.2–4.4)
All	7.6 (7.3–7.8)	5.5 (4.8–6.2)
Controls		
Mental disorders	2.3 (2.1–2.5)	−0.1 (−0.6–0.4)
Musculoskeletal diseases	3.3 (3.1–3.6)	0.4 (−0.2–0.9)
Other diseases	3.2 (3.0–3.4)	0.1 (−0.3–0.6)
All	3.0 (2.8–3.1)	0.1 (−0.2–0.4)

**Table 3:** Excess number of unemployment days among cases compared to the controls during the years preceding disability retirement by the study variables, mean with 95% confidence intervals adjusted for gender, age and calendar year. The last year before disability retirement omitted.

	Disability Retirees			
	All	Diagnosis of Disability Retirement		
		Mental Disorders	Musculoskeletal Diseases	Other Diagnoses
All	38.6 (37.8–39.4)	59.5 (58.0–60.9)	24.5 (23.1–25.9)	33.7 (32.4–35.0)
Gender				
Men	41.2 (40.1–42.3)	67.0 (64.8–69.2)	23.8 (21.8–25.8)	37.2 (35.5–39.0)
Women	35.7 (34.5–36.8)	52.9 (50.9–54.8)	25.1 (23.0–27.1)	28.3 (26.3–30.3)
Age				
25–34 years	46.2 (44.0–48.3)	49.4 (46.8–52.0)	36.2 (29.8–42.6)	41.3 (37.0–45.6)
35–44 years	52.3 (50.2–54.4)	71.6 (68.4–74.7)	31.3 (26.9–35.8)	35.9 (32.3–39.5)
45–54 years	45.8 (44.3–47.3)	67.2 (64.5–69.9)	31.5 (28.8–34.2)	38.5 (36.2–40.7)
55–64 years	29.7 (28.4–30.9)	50.4 (47.3–53.4)	20.4 (18.6–22.2)	30.1 (28.2–31.9)
Educational level				
Primary	36.7 (34.7–38.6)	64.0 (60.2–67.8)	19.7 (16.5–23.0)	32.8 (29.8–35.9)
Secondary	35.3 (34.1–36.5)	60.2 (58.0–62.4)	17.5 (15.4–19.5)	32.7 (30.8–34.7)
Lower tertiary	24.8 (23.4–26.3)	35.4 (33.0–37.8)	20.2 (17.0–23.5)	18.1 (15.8–20.5)
Higher tertiary	25.1 (22.7–27.4)	38.0 (34.5–41.5)	25.2 (17.7–32.8)	11.3 (7.6–15.0)
Occupational class				
Manual workers	17.1 (15.9–18.4)	40.7 (38.0–43.4)	5.1 (3.0–7.1)	16.5 (14.5–18.4)
Lower non-manual employees	21.9 (20.8–23.1)	33.6 (31.6–35.6)	17.3 (15.3–19.4)	16.0 (14.1–17.8)
Upper non-manual employees	20.3 (19.0–21.7)	27.6 (25.3–29.9)	27.4 (24.1–30.7)	11.6 (9.6–13.6)
Entrepreneurs	15.4 (14.2–16.7)	23.3 (20.6–26.0)	15.7 (13.5–17.9)	10.7 (8.9–12.5)
Other or unknown	69.8 (66.1–73.4)	57.6 (51.9–63.4)	86.4 (78.4–94.4)	71.9 (65.8–78.0)

**Table 4:** Average increase in the number of unemployment days per one year during the years before disability retirement by the study variables (mean and 95% confidence intervals) adjusted for gender, age and calendar year. The last year before disability retirement omitted.

	Disability Retirees			
	All	Diagnosis of Disability Retirement		
		Mental Disorders	Musculoskeletal Diseases	Other Diagnoses
All	5.5 (4.8–6.2)	8.0 (6.6–9.4)	3.8 (2.6–5.0)	3.3 (2.2–4.4)
Gender				
Men	6.3 (5.3–7.3)	8.8 (6.7–10.9)	4.7 (3.1–6.3)	4.2 (2.7–5.7)
Women	4.8 (3.8–5.8)	7.1 (5.3–9.0)	2.9 (1.2–4.6)	2.2 (0.5–3.9)
Age				
25–34 years	5.0 (2.7–7.2)	6.1 (3.2–8.9)	3.4 (−3.4–10.1)	1.1 (−3.4–5.6)
35–44 years	6.5 (4.3–8.6)	8.0 (4.7–11.3)	3.4 (−0.9–7.6)	3.4 (−0.1–6.9)
45–54 years	7.7 (6.2–9.1)	10.6 (7.8–13.5)	5.3 (2.7–7.9)	5.6 (3.3–7.8)
55–64 years	4.1 (2.9–5.2)	6.5 (3.4–9.6)	3.4 (1.8–5.1)	2.9 (1.1–4.7)
Educational level				
Primary	6.0 (4.5–7.4)	8.1 (5.2–11.1)	3.5 (1.1–5.8)	4.4 (2.1–6.7)
Secondary	5.2 (4.3–6.2)	7.1 (5.1–9.1)	3.8 (2.3–5.3)	3.0 (1.4–4.6)
Lower tertiary	3.6 (2.1–5.1)	5.4 (2.8–8.1)	3.3 (0.1–6.6)	1.2 (−1.0–3.5)
Higher tertiary	6.0 (3.2–8.9)	8.2 (3.7–12.7)	7.9 (−1.9–17.8)	1.6 (−2.1–5.2)
Occupational class				
Manual workers	7.7 (6.7–8.7)	12.0 (9.6–14.4)	4.5 (3.1–5.9)	6.8 (5.2–8.3)
Lower non-manual employees	6.0 (4.9–7.0)	8.3 (6.3–10.4)	4.5 (2.6–6.3)	4.1 (2.4–5.7)
Upper non-manual employees	5.7 (4.0–7.5)	8.4 (5.5–11.3)	8.2 (3.3–13.1)	2.1 (−0.3–4.4)
Entrepreneurs	3.0 (1.6–4.3)	4.9 (1.8–8.0)	2.3 (−0.1–4.7)	1.9 (0.1–3.8)
Other or unknown	5.6 (3.5–7.6)	4.7 (1.7–7.7)	14.3 (9.7–19.0)	3.6 (0.0–7.2)
